# Effects of a traditionally-dosed creatine supplementation protocol and resistance training on the skeletal muscle uptake and whole-body metabolism and retention of creatine in males

**DOI:** 10.1186/1550-2783-12-S1-P2

**Published:** 2015-09-21

**Authors:** Joshua J Gann, Sarah K McKinley-Barnard, Thomas L Andre, Ryan D Schoch, Darryn S Willoughby

**Affiliations:** 1 Exercise and Biochemical Nutrition Lab, Department of HHPR, Baylor University, Waco, TX 76798, USA

## Background

A typical oral creatine supplementation regimen involving a 5-7 day "loading phase" of 20-25 grams/day followed by a "maintenance phase" of 5-7 grams/day is typically considered as necessary to adequately saturate skeletal muscle as a lesser dose of creatine is insufficient in doing so. This rationale also assumes that the majority, if not all, of the creatine ingested at this dosage is fully utilized by skeletal muscle as a phosphate reservoir in which to re-synthesize ATP during high-intensity, short-term exercise. The purpose of this study was simply to determine the effects of this "typical" creatine dosing strategy previously mentioned on skeletal muscle creatine uptake as well as the whole-body metabolism and retention of creatine in males while engaged in resistance training.

## Methods

In a double-blind manner, fourteen (Cr = 7, Pl = 7) non-resistance-trained (i.e. < thrice weekly, 1 year prior) men between the ages of 18-30 were randomly assigned by age and body weight to orally ingest a powdered dextrose placebo or creatine monohydrate. After baseline strength and body composition testing procedures, participants ingested creatine or placebo at a dose of 0.3g/kg lean body mass/day (≈ 20-25g/day) for a 5 day loading phase immediately followed by a 42-day maintenance phase at a dose of 0.075g/kg lean body mass/day (≈ 5-7g/day). The participants followed a periodized 4 day per week resistance-training program split into two upper body and two lower body workouts per week, for a total of 7 weeks. Blood and muscle samples were obtained at Day 0, 6, 27, and 48. Statistical analyses were performed utilizing separate two-way ANOVA for each criterion variable employing a probability level of ≤ 0.05.

## Results

Creatine supplementation preferentially induced significant increments in total body mass (p = 0.03) and lean body mass (p = 0.01). Creatine did not significantly decrease fat mass (p = 0.29); however, fat mass was significantly decreased in both groups with resistance training (p = 0.001). Muscle strength significantly increased with resistance training (p = 0.001) for both groups, but was not preferentially increased with creatine supplementation. Creatine supplementation significantly increased muscle total creatine (p = 0.043), serum creatine (p = 0.003), urinary creatine (p = 0.036), and urinary creatinine (p = 0.01) in the creatine group compared to placebo.

## Conclusion

A typically-dosed creatine supplementation regimen produced increases in total and lean body mass, despite the inability to preferentially increase muscle strength in conjunction with resistance training. This regimen was also able to effectively increase muscle total creatine content; however, this dosing strategy for creatine supplementation also led to excess amounts of serum and urinary creatine and urinary creatinine content. Despite increases in body mass and muscle creatine uptake with this regimen, a similar response may likely occur with a lesser creatine dose in non-resistance trained males.

